# A141 IS SURGERY STILL THE ONLY TREATMENT OPTION FOR CURABLE RECTAL CANCER?

**DOI:** 10.1093/jcag/gwab049.140

**Published:** 2022-02-21

**Authors:** T Vuong, A Garant, F Khosrow-Khavar, S Devic, S Enger, M Boutros, A Cohen, C S Miller, G Friedman, P Galiatsatos, V Nguyen, N Benoit, H Lan Thai, H Diec, C Desgroseilliers, J Faria, C Vasilevsky

**Affiliations:** 1 Radiation Oncology, Sir Mortimer B Davis Jewish General Hospital, Montreal, QC, Canada; 2 Medicine, Division of Gastroenterology, SMBD Jewish General Hospital, Montrreal, QC, Canada; 3 Hopital Pierre-Boucher, Longueuil, QC, Canada; 4 The University of Texas Southwestern Medical Center, Dallas, TX

## Abstract

**Background:**

Rectal cancer is curable by standard surgery with Total Mesorectal Excision (TME). However, there are well known associated long-term bowel and sexual dysfunctions. Non-operative management (NOM) is an emerging treatment for patients with operable rectal cancer. There is evidence supporting dose response for tumor control in rectal adenocarcinoma.

**Aims:**

In the era of modern technologies, Image-guided adaptive endorectal brachytherapy is a means to deliver local radiotherapy boost treatments. We explored its role in a randomized phase II/III trial (NCT03051464) for patients aiming to achieve cure without surgery. Total Mesorectal Excision (TME) free survival at 2 years was the primary endpoint. We now present the interim analysis upon accrual of the first 40 patients.

**Methods:**

In randomized trial, patients with operable cT2-3ab N0 M0 rectal cancer received 45 Gy in 25 fractions of pelvic external beam radiotherapy (EBRT) with concurrent 5-FU/ Capecitabine. They were randomized to receive either an EBRT boost of 9 Gy in 5 fractions (Arm A), or three weekly adaptive brachytherapy boosts for a total of 30 Gy in 3 fractions (Arm B).

**Results:**

Forty patients were included (20 per arm). The median age was 66 years; baseline characteristics were well balanced in terms of age, tumor location, T stage and tumor size (Table 1). The acute treatment related toxicities are similar as shown in table 2 but in arm B, there were two deaths: one patient died during his chemotherapy and external beam treatment from congestive heart failure and one patient from a heart attack after treatment prior to salvage TME surgery. The proportion of complete clinical response was 50% (n=10/20) in Arm A and 90% in Arm B (n=18/20). With a median follow-up of 2.2 years, local regrowth at 2 years occurred in 4/10 patients (40%) in Arm A and 4/18 patients (22%) in Arm B. TME-free survival rate at 2 years was 45.9% in Arm A and 85.1% in Arm B (p=0.0036) (Figure 1).

**Conclusions:**

The interim analysis of this trial suggests that these two strategies of radiation dose escalation are feasible and lead to high chances of organ preservation in patients with operable rectal cancer. The Independent Monitoring Comittee (IDMC) approved the continuation of patient recruitment in the phase III study as planned.

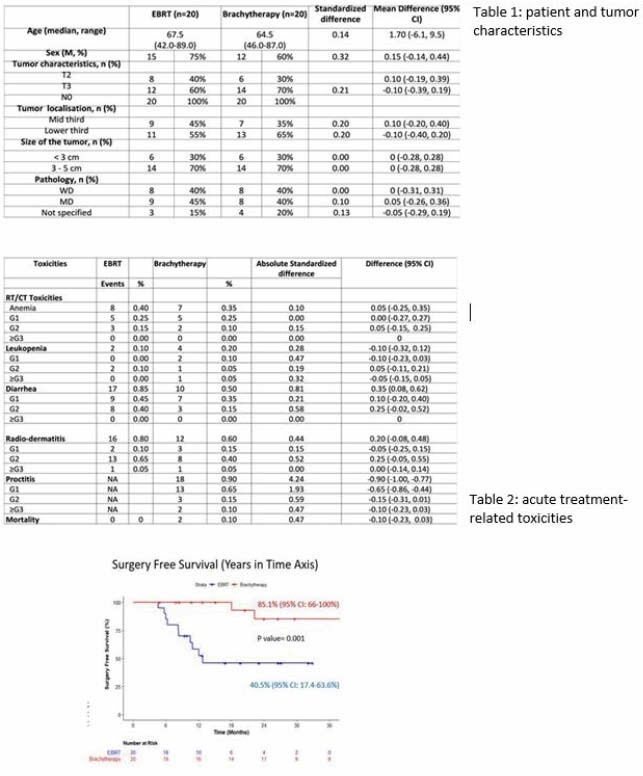

**Funding Agencies:**

Elekta

